# CXCL12 Modulates Prostate Cancer Cell Adhesion by Altering the Levels or Activities of *β*1-Containing Integrins

**DOI:** 10.1155/2014/981750

**Published:** 2014-12-15

**Authors:** Mehdi Dehghani, Sedigheh Kianpour, Ana Zangeneh, Zohreh Mostafavi-Pour

**Affiliations:** ^1^Department of Biochemistry, Shiraz University of Medical Sciences, Shiraz 71348-14336, Iran; ^2^Division of Oncology, Department of Internal Medicine, University of Texas Health Science Center, Houston, TX 77030, USA; ^3^School of Public Health, University of Texas Health Science Center, Houston, TX 77030, USA; ^4^Recombinant Protein Laboratory, School of Advanced Medicinal Sciences and Technologies, Shiraz University of Medicinal Sciences, Shiraz 71348-14336, Iran

## Abstract

The mechanisms by which prostate cancer (PCa) cell adhesion and migration are controlled during metastasis are not well understood. Here, we studied the effect of CXCL12 in PCa cell adhesion and spreading in DU145 and PC3 cell lines using as substrates collagen I, fibronectin (FN), and their recombinant fragments. CXCL12 treatment increased *β*1 integrin-dependent PC3 cell adhesion on FN which correlated with increased focal adhesion kinase activation. However neither *α*5*β*1 nor *α*4*β*1 subunits were involved in this adhesion. By contrast, CXCL12 decreased DU145 adhesion and spreading on FN by downregulating *α*5 and *β*1 integrin expression. To demonstrate the clinical relevance of CXCL12 in PCa, we measured CXCL12 levels in plasma by using ELISA and found that the chemokine is elevated in PCa patients when compared to controls. The high concentration of CXCL12 in patients suffering from PCa in comparison to those with benign disease or healthy individuals implicates CXCL12 as a potential biomarker for PCa. In addition these data show that CXCL12 may be crucial in controlling PCa cell adhesion on fibronectin and collagen I, possibly via crosstalk with integrin receptors and/or altering the expression levels of integrin subunits.

## 1. Introduction

Prostate cancer (PCa) is the most common cancer in men, with the highest reported cases among African-Americans. White men have the second highest rate of developing PCa, followed by Hispanic, Asian, and Native American men. In 2013, an estimated 238,590 new cases of PCa were reported in the US with about 29,720 deaths [1].

The mortality rate is mainly attributed to the spread of malignant cells to different organs including, but not limited to, bone, brain, and lymph nodes. Therefore, there is an increasing interest not only in the early detection and diagnosis of PCa, but also in unraveling the mechanisms that lead to metastasis [2]. Over the course of decades, the prostate-specific antigen (PSA) has allowed for the detection of PCa in its early stages. Despite its apparent increase in the detection of PCa, controversy regarding the efficacy of PSA as a tumor marker exists. The success rate for an early detection and diagnosis depends heavily on clinical biomarkers; however, the current biomarker used for PCa is not ideal; thus there is a need for more reliable indicator to determine the correct treatment for patients [3].

Metastasis is a multistep process by which invasive capable cells disseminate from primary tumor lesions to other organs through several steps of cell-cell and cell-extracellular matrix (ECM) attachments and detachments [4]. Despite the known detrimental effects of cancer metastasis, this process still remains elusive at both cellular and molecular levels. Integrins which transmit both mechanical and chemical signals are cell-surface receptors that bind to ECM components and thereby affect cell cytoskeleton rearrangement and intracellular signaling pathways. Integrins are heterodimers of *α* and *β* subunits of which 8*β* subunits can assort with 18*α* to form 24 integrins which bind to distinct subsets of ECM ligands [5]. Fibronectin and collagen-integrin interaction play an important role in tumor cell migration and metastasis [6–9]. Chemokines, a superfamily of small molecular weight chemoattractant cytokine, are among the factors that affect cancer cell invasion and metastasis by changing cytoskeletal rearrangement, cell adhesion to ECM proteins and endothelial cells, and directional migration [10]. Among the chemokines, CXCL12, also known as stromal cell derived factor-1 (SDF-1), and its cognate receptor CXCR4 have been involved in cancer metastasis of several cancers where the CXCL12-CXCR4 axis is known to modulate phenomena such as chemotaxis, migration, proliferation, and angiogenesis [11, 12]. This axis has been shown to modulate the expression and activity of integrin receptors in renal cell carcinoma (RCC) [13]. The role of CXCL12 in the directional metastasis of PCa to bone has been reported [14, 15]. Histopathological analysis of human tissues has shown that CXCR4 expression is absent or insignificant in normal prostate epithelial cell lines, but its expression is higher in cell lines that are used in PCa research (i.e., LNCaP, PC3) [10]. The primary objective of our study was to investigate whether plasma levels of CXCL12 in PCa patients are significantly different from controls and individuals suffering from benign prostatic hyperplasia (BPH). The second objective was to study the effects of CXCL12 on *β*1-containing integrin-dependent PCa cell adhesion.

## 2. Materials and Methods

### 2.1. Materials

FITC-conjugated mouse anti-human CD49e that reacts with *α*5 chain of VLA-5 complex (*α*5*β*1 integrin), FITC-conjugated IgG2 negative control for *α*5 subunit from *α*5*β*1 integrin, FITC-conjugated mouse anti-human CD49d that recognizes *α*4 subunit of VLA-4 complex (*α*4*β*1 integrin), FITC-conjugated mouse IgG1 negative control for *α*4 and *β*1 subunits, and FITC-conjugated mouse anti-human CD29 antibody that recognizes the *β*1 subunit of human integrins were purchased from Serotec (UK).

Human recombinant CXCL12, Quantikine human CXCL12/SDF-1 immunoassay kit, and mouse anti-human CXCR4 were from R&D Systems (Minneapolis, USA). Mouse anti-human vinculin, TRITC-conjugated goat anti-rat IgG, and FITC-conjugated donkey anti-mouse IgG were purchased from Jackson (Immune Research Laboratories, USA) and rhodamine-conjugated phalloidin, COL-I, and human plasma FN were purchased from Sigma (USA). Mouse anti-human FAK, rabbit polyclonal anti-human p-FAK (Tyr 397), and goat anti-mouse and goat anti-rabbit IgG-HRP were purchased from Santa Cruz Biotechnology (USA). Anti-human mouse monoclonal *α*2*β*1 antibody (clone BHA2.1) was from Chemicon (USA).

Rat anti-human integrin *β*1 monoclonal antibody mAb 13, mouse anti-human integrin *α*4 monoclonal antibody HP2/1, mouse anti-human integrin *α*5 monoclonal antibody JBS5, the H/120 variant fragment of human FN that encompasses type III repeats 12–15 of the FN and the 50K fragment of FN (comprising FN type III repeats 6–10) were gifts from Dr. Martin Humphries (Center for Cell Matrix Research, University of Manchester, Manchester, UK).

### 2.2. Patients and Plasma Collection

After obtaining IRB approval and written informed consent, plasma samples were collected from 39 patients with untreated PCa (median age 71 years), 40 patients with benign prostatic hyperplasia (BPH) (median age 70 years), and 33 healthy individuals (median age 73 years) at Shiraz University of Medical Sciences Hospital between 2005 and 2007. Neither patients nor controls had apparent severe infection or autoimmune diseases. PCa as well as BPH diagnosis was confirmed pathologically by transrectal ultrasonography guided systematic biopsy. The patient tumors were categorized according to their Gleason score which ranged from 4 to 10 PSA levels and had been measured by radioimmunoassay (RIA) method in the diagnostic lab of the above-mentioned hospital.

### 2.3. ELISA

Plasma levels of CXCL12 in age-matched healthy individuals and patients suffering from PCa and BPH were measured by Quantikine human CXCL12/SDF-1 immunoassay kit (R&D Systems, Minneapolis, USA) according to the manufacturer's instructions. Analyses and calibrations were carried out in duplicate and intra- and interassay variations were within the range given by the manufacturer.

### 2.4. Cell Culture

PC3 and DU145 are two human metastatic PCa cell lines which have been established from a metastatic lesion to bone and brain, respectively (National Cell Bank of Iran), and were cultured in RPMI 1640 medium containing 10% fetal bovine serum (FBS) and penicillin/streptomycin at 37°C in a humidified atmosphere containing 5% CO_2_. Cells were grown to 80% confluence and then starved in media containing 0.5% FBS overnight prior to stimulation.

### 2.5. Cell Spreading Assay

This assay was performed in the adhesive coated 96-well plates. Cells were detached and resuspended in RPMI 1640 medium without any additives and treated with mock buffer or stimulant. Aliquots of cell suspension (100 *μ*L) were immediately added to substrate-coated wells and incubated in a humidified air atmosphere for 90 minutes. Cells were then fixed by addition of 50% (w/v) glutaraldehyde for 30 minutes at room temperature. Glutaraldehyde was carefully aspirated; then PBS containing 0.02% (w/v) sodium azide was added until an inverted meniscus was formed at top of each well. Glass coverslips were applied onto the 96-well tissue culture plate, and the percentage of cell spread in each well was determined by phase contrast microscopy. A total of 400 cells/well from a number of randomly selected fields were counted. The criteria for a spread cell included a phase dark appearance and an area visible cytoplasm around the nucleus.

### 2.6. Cell Attachment Assay

This assay was performed in the adhesive coated 96-well plates as discussed previously [16, 17]. First, cells were detached and resuspended in RPMI 1640 medium without any additives and treated with mock buffer, stimulant, normal control antibodies, and/or inhibitory anti-integrin antibodies; they were then loaded into the coated wells and incubated for 20 minutes; then unbound or loosely bound cells were washed off by aspiration and mild washing with PBS. Cells were fixed by 5% glutaraldehyde in PBS. In order to measure the total number of cells per well, 100%, 75%, 50%, 25%, and 0% cells were seeded into the wells and fixed by the addition of 50% (v/v) glutaraldehyde 1 : 10. Wells were aspirated and washed with PBS before addition of 0.1% (w/v) crystal violet in methylethanesulphonic acid (MES) pH6 for 60 minutes. Wells were then aspirated and washed with distilled water before the addition of 10% (v/v) acetic acid. At the end the absorbance of each well was measured with a multiscan plate reader at 570 nm.

### 2.7. Immunofluorescence

Immunofluorescence was done as discussed before [17]. Glass coverslips (13-mm diameter) were coated with FN or COL-I diluted in PBS; then 10 mg/mL heat-denatured BSA was used to inhibit nonspecific binding. Cells were detached and suspended in RPMI 1640 media without serum (4 × 10^4^ cells/mL); then 0.5 mL aliquots of cells were added onto the coverslips and incubated for 2 h at 37°C. Then cells were fixed and permeabilized with Triton X-100 diluted in PBS, washed with PBS, and blocked by 3% BSA solution. The cells were immunostained with primary and appropriate secondary antibodies diluted in blocking buffer. F-actin was detected using rhodamine-conjugated phalloidin (1 : 1,000 dilution; Sigma-Aldrich) in blocking buffer. Coverslips were mounted face down on glass slides using 5 *μ*L Vectashield (Vector Laboratories) and observed using a microscope (Olympus); images were taken in the green and red channels using a CCD camera.

### 2.8. RNA Extraction and Semiquantitative Reverse Transcriptase Polymerase Chain Reaction (RT-PCR)

The messenger RNA (mRNA) expression levels of *β*1, *α*2, *α*4, and *α*5 integrin subunits were evaluated by RT-PCR as discussed before [18, 19]. Total RNA was extracted from DU145 and PC3 cells using Tripure isolation reagent (Roche Applied Science, Germany), according to the manufacturer's instructions. RT-PCR experiments were carried out with cDNAs produced from 1 *μ*g of extracted RNA using first strand cDNA synthesis kits (Fermentas, Germany). The PCR products were resolved on 1.5% agarose gel electrophoresis. The RT-PCR products corresponding to integrins and glyceraldehyde-3-phosphate dehydrogenase (GAPDH) cDNAs were detected as a single band of the expected size. The level of cDNA was evaluated by densitometry using UVIDoc software version 15.

### 2.9. Flow Cytometry

To analyze integrin surface expression, 50 *μ*L of cells was incubated on ice with 50 *μ*L FITC-conjugated monoclonal antibodies, anti-*α*4, anti-*α*5, and anti-*β*1 integrins (or FITC-conjugated mouse IgG), diluted in blocking buffer (PBS and 1% BSA) for an hour. Cells were washed three times with PBS and fixed in 0.4% formaldehyde. The cells were analyzed on a FACS instrument (BD Biosciences, USA).

### 2.10. Focal Adhesion Kinase (FAK) Activation

Starved cells were stimulated with CXCL12 (200 ng/mL) and lysed in RIPA buffer containing phosphatase and protease inhibitors; then lysates were heated up to 95°C for 5 min, separated on a 4% to 12% Bis-Tris NuPAGE gel (Invitrogen), and transferred to nitrocellulose for immunoblotting. Blots were incubated with primary and secondary antibodies against pFAK and FAK, and protein signals were detected with Novex ECL Western blotting detection reagents (Invitrogen).

## 3. Results and Discussion

### 3.1. Elevated Plasma CXCL12 Levels in Human PCa

CXCL12 is commonly expressed in various organs such as the heart, liver, kidney, and skeletal muscle. However, vascular endothelial cells, osteoblasts, and stromal fibroblasts are major cellular sources of this chemokine. High levels of CXCL12 have been reported in several human cancers [12]. A strong correlation exists between CXCL12 expression and breast cancer metastasis to bone marrow and lymph nodes [20]. Plasma levels of this chemokine were shown to be significantly higher in breast cancer patients than in age-matched controls and had a significant correlation with tumor grade [21].

Because reported data regarding the measurement of circulating CXCL12 levels in patients with PCa is scarce, the plasma levels of CXCL12 in patients with PCa were measured in order to evaluate any increase in systemic levels compared to BPH and age-matched controls. The levels of CXCL12 in three groups (BPH, *n* = 40; PCa, *n* = 39; and controls, *n* = 33) were compared (Table 1). The Kruskal-Wallis and the Wilcoxon signed rank tests showed that the median level of CXCL12 was significantly higher in PCa (*P* < 0.0001). The Tukey multiple test showed PCa patients to have significantly higher mean differences (*P* < 0.001). The Spearman test determined a positive correlation between plasma CXCL12 level and the reported Gleason scores of PCa patients (*P* < 0.01). In order to compare CXCL12 level and the stage of cancer, PCa patients were divided into two subgroups according to their Gleason scores. Because a Gleason score of 4 + 3 is a more aggressive cancer than a Gleason score of 3 + 4, the following two subgroups were determined: <7 including 3 + 4 (subgroup L) and >7 including 4 + 3 (subgroup H). PCa patients of 4 + 3 were associated with a threefold increase in lethal PCa compared to 3 + 4 cancers [22]. The *t*-test analysis and the Wilcoxon signed rank test showed that CXCL12 plasma concentrations in subgroup H were significantly higher (Table 2). Reported plasma PSA concentration levels of PCa and BPH patients were 5.8 to 100 ng/mL and 3.0 to 75.0 ng/mL, respectively. The mean PSA levels in PCa patients (24.5 ± 9.3 ng/mL) were significantly higher than those of BPH patients (16.2 ± 11.17 ng/mL) (*P* < 0.03). The median PSA levels for PCa and BPH patients were 15.5 and 10.9, respectively. A linear correlation between circulating levels of PSA and CXCL12 was not observed. In agreement with a study by Macoska et al. [23], we found that PCa patients (*n* = 9) with PSA levels <10 ng/mL had significantly higher mean and median CXCL12 levels (1.85 ± 0.38) than BPH patients (*n* = 16) with PSA levels <10 ng/mL (1.46 ± 0.3) (*P* < 0.01). No correlation was found between CXCL12 levels and the age of patients. Overall the results of this study show that plasma CXCL12 levels in PCa are elevated and may potentially be used to distinguish between BPH and PCa in patients with serum PSA levels lower than 10 ng/mL.

### 3.2. CXCL12 Modifies PCa Cell Adhesion on FN and COL-I

There is emerging evidence that the CXCR4-CXCL12 axis regulates directional migration and metastasis in a variety of cancers [12]. CXCL12-CXCR4 interactions have been shown to play a role in the metastasis of PCa to bone [14, 15, 24]; however, there is sparse information on the roles of this chemokine along with the integrins involved in PCa cell adhesion, particularly the way CXCL12 regulates cell-ECM interactions.

### 3.3. PC3 and DU145 Cell Lines Adhere to FN and COL-I

FN and COL are the main ECM proteins that physically connect the cells to the adjacent substrata through interactions with corresponding integrin receptors [9].

**Figure 1:**
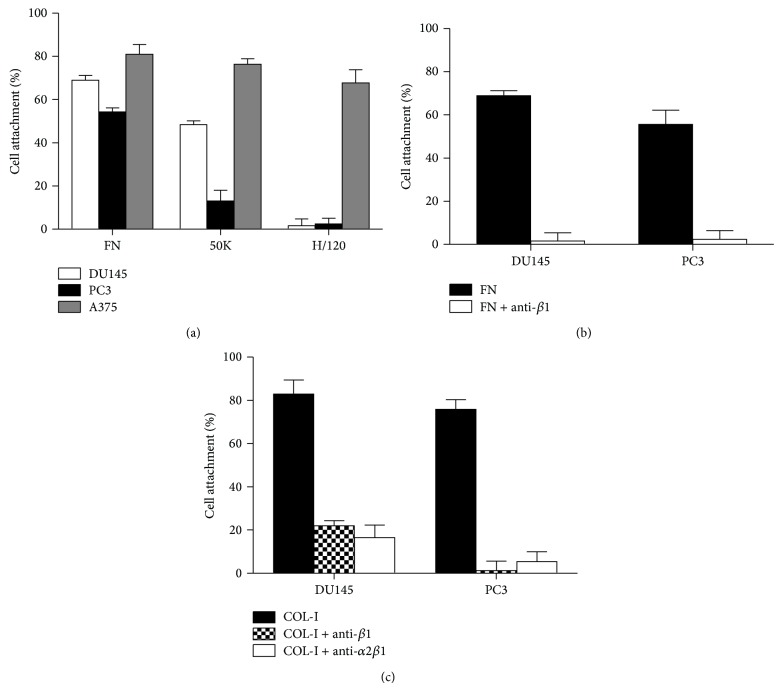
(a) PC3 and DU145 cell attachments on FN, 50K, and H/120. A375 cell was used as a control; A375 is positive for both *α*4*β*1- and *α*5*β*1-mediated cell adhesion on FN. (b) The effect of anti-*β*1 integrin antibody on DU145 and PC3 cells attachment to FN (10 *μ*g/mL). (c) The effect of anti-*β*1 and anti-*α*2*β*1 integrin antibodies (10 *μ*g/mL) on DU145 and PC3 cell attachment to COL-I (5 *μ*g/mL). The level of nonspecific binding, determined from these cells attachment to wells coated with BSA alone, was subtracted. Mouse and rat normal control antibodies have no impact on the levels of DU145 and PC3 cell attachment to FN and COL-I. Values shown are mean ± standard deviation of triplicate wells.

To determine the involvement of probable integrins in PC3 and DU145 cell adhesion to ECM, the ligands COL-I, FN and two different recombinant fragments of FN, 50K, and H/120 were tested (Figure 1(a)). The 50K and H/120 are recombinant fragments of FN that specifically bind to *α*5*β*1 and *α*4*β*1, respectively [17]. As shown in Figure 1(b), 54.4 ± 1.3 and 70.8 ± 2.5 percent of seeded PC3 and DU145 cells attach on FN, respectively. More than 95% of these adhesions were suppressed in the presence of anti-*β*1 integrin antibody, mAB 13. Rat normal control antibody had no effect on the adhesion levels of both cell lines on FN. These data reveal that PC3 and DU145 cells adhere on FN using *β*1-containing integrins. To investigate whether *α*5*β*1 and/or *α*4*β*1 integrins are involved in this adhesion, these cells were cultured onto the recombinant 50K and H/120 fragments. Minor percentages of PC3 and DU145 were able to attach to H/120; however, both DU145 and PC3 cells attached on 50K fragment of FN (Figure 1(a)). A375 cell which was previously [17] shown to have *α*4*β*1 and *α*5*β*1 integrin-dependent cell adhesion on FN and its recombinant fragments (H/120 and 50K) was used as a positive control. These data revealed that both DU145 and PC3 cells do not use *α*4*β*1 integrin to attach to FN; however, DU145 cell adhesion on FN is mainly mediated by *α*5*β*1 and PC3 cell adhesion on FN may be slightly mediated by *α*5*β*1 with the possibility of other *β*1-containing integrins in the process.

As depicted in Figure 1(c), PC3 and DU145 cell adhesion onto COL-I are significantly inhibited by either anti-*β*1 (clone mAB 13) or anti-*α*2*β*1 integrin antibodies (clone BHA2.1), which indicate that this adhesion is predominantly *α*2*β*1 dependent. Additionally, spiking normal control antibodies did not have any effect on the cell adhesion levels of the cells on COL-I.

### 3.4. CXCL12 Affects PC3 and DU145 Cells Adhesion and Focal Adhesion Formation on FN and COL-I

**Figure 2:**
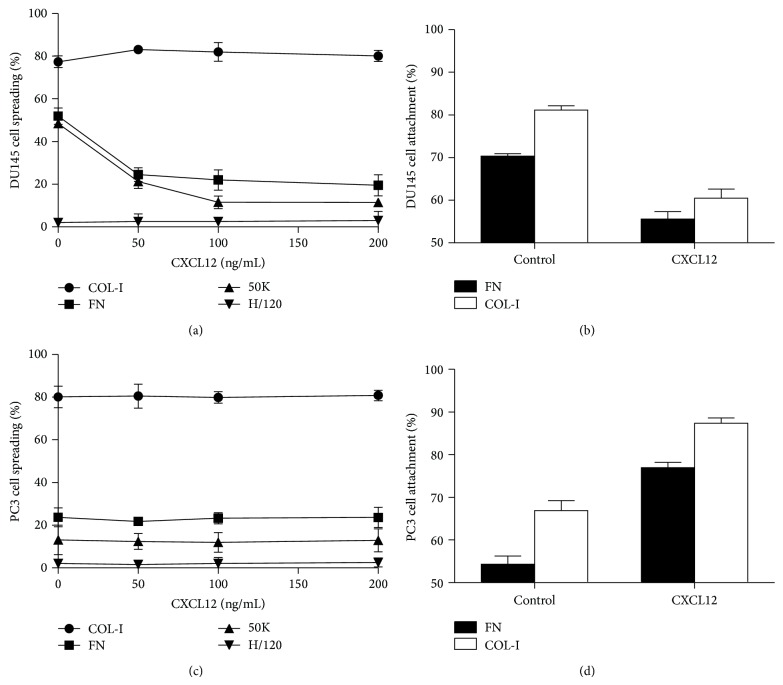
(a) DU145 cell spreading on COL-I, FN, 50K, and H/120 fragment of FN in the presence of various concentrations of CXCL12 (200 ng/mL). (b) Attachment of DU145 cells on FN and COL-I in the absence and presence of CXCL12 (200 ng/mL). (c) PC3 cell spreading on COL-I, FN, 50K, and H/120 fragment of FN in the presence of various concentrations of CXCL12 (200 ng/mL). (d) PC3 cell attachment on FN and COL-I in the absence and presence of CXCL12 (200 ng/mL). The level of nonspecific binding, determined from these cells attachment to wells coated with BSA alone, was subtracted. Values shown are mean ± standard deviation of triplicate wells.

**Figure 3:**
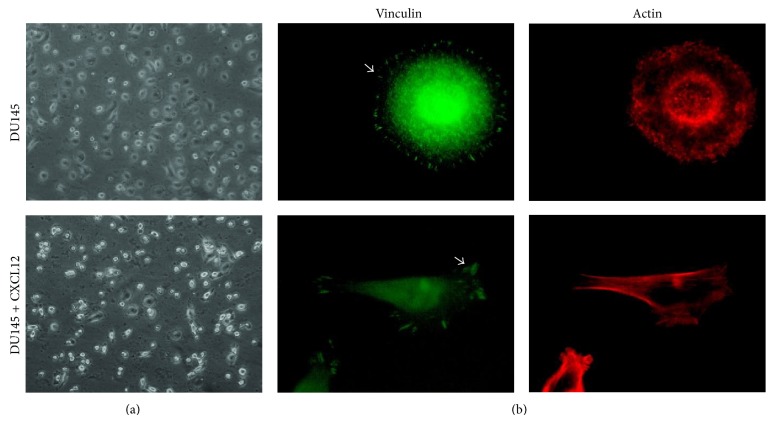
CXCL12 induces focal adhesion disassembly, actin stress fiber rearrangement, and morphological change in DU145 cells seeded on FN. Cells were incubated for 2 hrs, fixed, and double-stained for vinculin and actin. Arrows in the images indicate localization of vinculin at the end of actin bundles in focal adhesion complexes.

Chemokines which are involved in the chemotaxis of lymphocytes are among the factors that assist in the dissemination of cancer cells from primary tumor lesions and landing at specific secondary sites to promote organ-specific metastasis [12]. Though there are a few studies on the role of CXCL12 on PC cell adhesion by integrins [25, 26], the effects of this chemokine on *β*1-containing integrin-dependent cell adhesion on FN and COL have yet to be explored. There have also been no reports to date concerning the effects of CXCL12 on PC3 cell adhesion-mediated by integrins on these ECM proteins. Considering that PC3 is derived from PCa metastasis to bone [27], one of the major sources of CXCL12, it would prove to be more relevant to test and compare the effect of CXCL12 on PC3 cell with DU145. It should be noted that DU145 is a PCa cell line derived from a human prostate adenocarcinoma brain metastasis [27]. To determine the effect of CXCL12 on integrin-mediated cell adhesion, PC3 and DU145 cells were seeded onto FN in the presence of various concentrations of CXCL12 (0–200 ng/mL). As shown in Figure 2(a), when DU145 cells were treated with CXCL12, the percentage of spread cells decreased significantly and refractile morphological changes and cell detachment were noticed; while some cells appeared round, other cells became elongated and spindle-shaped, resembling mesenchymal cells (Figure 3(a)). Consistent with cellular morphological changes, dramatic actin reorganization and redistribution of the focal adhesion protein vinculin were observed (Figure 3(b)). Vinculin is involved in stabilizing focal adhesion complex by regulating integrin clustering [28, 29]. Focal adhesions are large, dynamic protein assemblies containing integrin clusters and several signaling molecules through which the cytoskeleton of a cell anchors to the ECM [28, 30]. Focal adhesions in round cells were not apparent, while elongated cells revealed a few lamellipodia; overall, CXCL12 treatment increased focal adhesion disassembly. The ability of CXCL12 to modify DU145 cell attachment on FN was also tested (Figure 2(b)). In agreement with cellular morphological changes, this chemokine caused a significant decrease in cell attachment (*P* < 0.05). These data are in conflict with results reported by Engl et al. which showed that CXCL12 increased the attachment of DU145 on FN [26].

**Figure 4:**
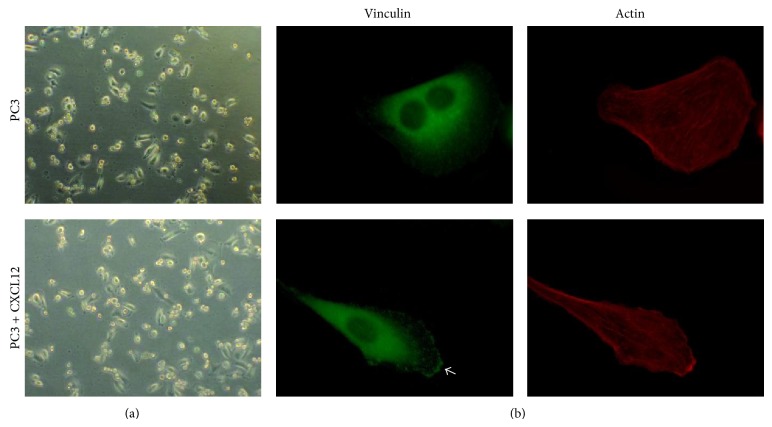
CXCL12 induces focal adhesion formation, actin stress fiber rearrangement, and morphological change in PC3 cells seeded on FN. Cells were incubated for 2 hrs, fixed, and double-stained for vinculin and actin. Arrows in the images indicate localization of vinculin at the end of actin bundles in focal adhesion complexes.

After treating PC3 cells with CXCL12, the percentage of spread cells did not change considerably (Figure 2(c)); however, a higher percentage of fully spindle-shaped PC3 cells were observed (%10.7 ± 2.4 compared to %24.3 ± 1.5; Figure 4(a)). PC3 cell attachment on FN was also significantly increased (Figure 2(d)). The ability of CXCL12 in modulating focal adhesion formation and microfilament polymerization in PC3 cells seeded on FN were also assessed. The CXCL12-treated PC3 cells showed higher numbers of organized stress fibers and focal contacts than untreated PC3 cells. CXCL12 also stimulated lamellipodia formation and actin stress fiber rearrangements (Figure 4(b)).

The effects of CXCL12 on *α*2*β*1 integrin-dependent PC3 and DU145 cell adhesion onto COL-I were also tested. This chemokine increased PC3 cell (Figure 2(d)) attachment and greatly decreased DU145 (Figure 2(b)) cell attachment on COL-I. PCa cells can adhere and proliferate on COL-I, which is abundant in bone [31]. This adherence as well as proliferation of PCa cells was shown to be mediated by *α*2*β*1 integrins [32]. The stimulatory effect of CXCL12, which is highly expressed in bone, on PC3 cell adhesion on COL-I may play a role in the ability of this cell line to metastasize to bone.

**Figure 5:**
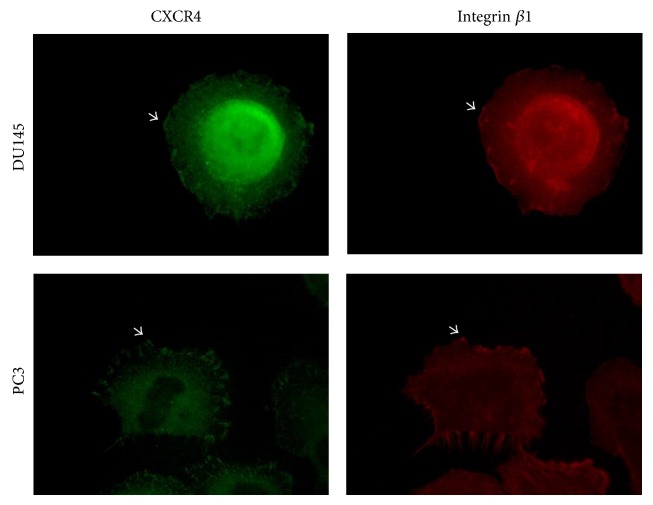
CXCR4 and integrin *β*1 are located closely at focal contact sites. PC3 and DU145 cells were incubated for 2 hrs, fixed, and double-stained for CXCR4 and *β*1 integrin. Arrows in the images indicate the localization of both CXCR4 with *β*1 integrin in focal contacts.

Overall, these findings indicate that CXCL12 modifies PC3 and DU145 *β*1-integrin mediated cell adhesion differently. In order for a cancer cell to migrate, it requires an intermediate level of attachment to be able to generate adequate traction force [4, 5]. For instance, EGF can induce either assembly or disassembly of focal adhesion complexes in a cell-dependent manner causing increased cell motility [33]. Without doubt, CXCL12 through CXCR4 activation increases PC cell migration as noted in other studies [2, 10, 15, 24, 34, 35]. The CXCL12-CXCR4 axis by integrin activation also resulted in an increased adhesion of small cell lung cancer (SCLC) cells to FN and COL [36–38]. Considering the high levels of CXCR4 expression on DU145 and PC3 cells [2, 15], we examined whether CXCR4 is closely localized within *β*1 integrin containing adhesion structures using a double-labeling immunofluorescence experiment for *β*1 integrin and CXCR4. As shown in Figure 5, there is a higher density of CXCR4 at *β*1 integrin containing focal contacts. This physical proximity may possibly be essential for the cooperation between CXCR4 and integrin receptors. The significance of the crosstalk between integrins and cytokine receptors on cancer cell adhesion and migration has been addressed previously [4, 5, 39].

### 3.5. CXCL12 Induces FAK Phosphorylation at Tyr 397

**Figure 6:**
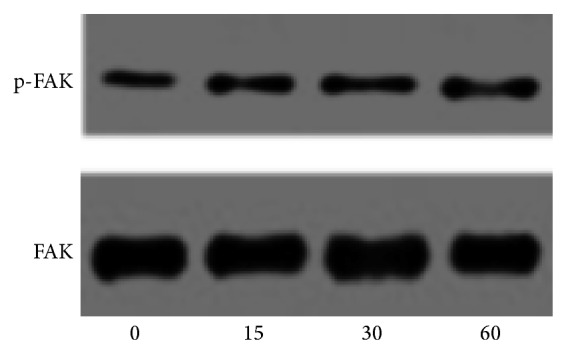
PC3 cells, untreated (0) or treated with CXCL12 (200 ng/mL), were lysed and then cell lysates were analyzed by immunoblotting with anti-tyrosine-phosphorylated site-specific antibody against FAK. The total protein was detected by probing the blots with anti-FAK (bottom panel). The blot was performed three times and a representative one is shown.

FAK, a nonreceptor tyrosine kinase, is a key player in crosstalk between growth factor receptors and integrins. FAK contains various tyrosine-containing motifs which upon phosphorylation interact with other signaling molecules such as src-related kinases, PI 3-kinase, the tyrosine phosphatase SHP2, and adaptor proteins Grb2 and Shc [40]. Following phosphorylation at Tyr 397, FAK indirectly binds to the cytoplasmic tail of *β*1 integrin subunit and by recruiting the involved signaling proteins to the sites of integrin receptor clustering stimulates cell movement [41, 42]. In most metastatic cancer cells, altered integrin expression profile and/or integrin activation are associated with downstream FAK phosphorylation [4]. The pivotal role of FAK phosphorylation along with *β*1 integrin in PCa migration has been noted previously [43]. A study by Shi and Boettiger has shown that the level of FAK phosphorylation at Tyr 397 is directly correlated to the number of integrin-fibronectin bonds [44]. In our study it was observed that CXCL12 (200 ng/mL) induces the phosphorylation of FAK at Tyr 397 in PC3 cells which is correlated with the morphological changes induced by CXCL12. The phosphorylated level of FAK increased 2.2 ± 0.17 times (*P* < 0.05) after 30 min stimulation by CXCL12 (Figure 6). Thus far, several studies have cited the involvement of FAK activation in CXCL12-induced integrin activation leading to increased cell invasion and migration [12, 13, 25, 26, 38].

### 3.6. Expression of Different Integrins on PC3 and DU145 Cells

Cell-dependent alteration of integrin repertoire provides the cancer cell the capability to not only disseminate to other organs, but also tolerate different environments [5, 45]. In human PC biopsies, the immunohistochemical levels of *α*3, *α*4, and *α*5 integrin subunits were found to be downregulated. Although downregulation of *α*2 subunits was also noted in 70% of grade II and III prostate adenocarcinomas, its level was found to be elevated in metastatic disease [46].

**Figure 7:**
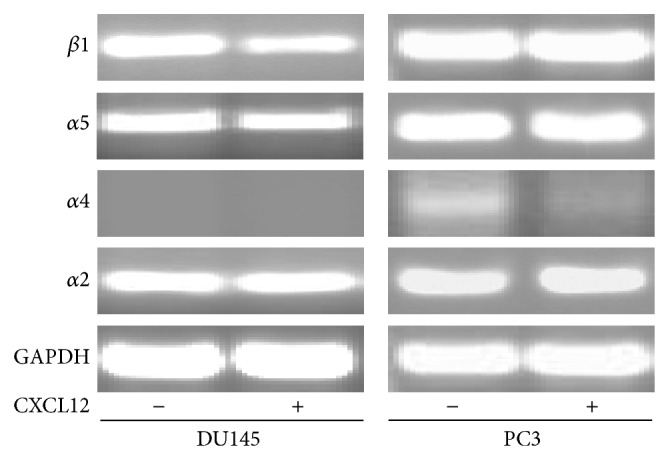
Ethidium bromide stained agarose gel electrophoresis of RT-PCR products of *α*2, *α*5, *α*4, and *β*1 integrin subunits in untreated and treated DU145 and PC3 cells.

**Figure 8:**
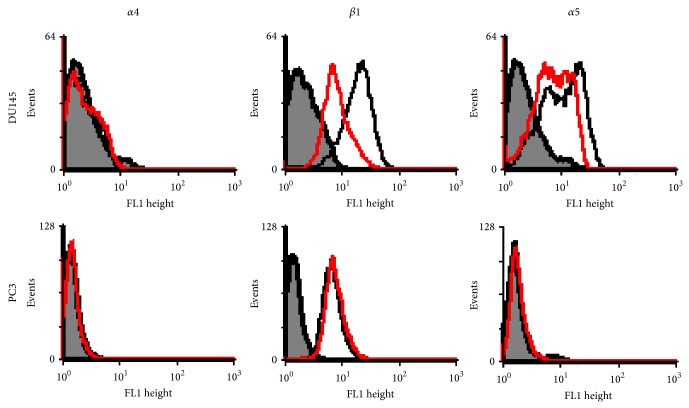
Flow cytograph analysis of *α*4, *β*1, and *α*5 integrin subunits in untreated (C curve) and 200 ng/mL CXCL12 (T curve) treated cells. The T curves represent the binding of anti-integrin antibody on treated cells, the C curves represent the binding of anti-*α*5 integrin antibody on untreated cells, and the mock curves (M) show the corresponding negative control antibody.

In this study the relevant integrin subunits' expressions were examined by semiquantitative RT-PCR and flow cytometry techniques. DU145 and PC3 cells showed considerable mRNA levels for *β*1, *α*2, and *α*5 integrin subunits. Among these genes, *α*4 integrin RNA expression was absent to very minor in DU145 and PC3, respectively. The effects of CXCL12 on integrin subunits' expression were also studied, even though the accuracy of results should be confirmed by quantitative RT-PCR. As shown in Figure 7, CXCL12 (200 ng/mL) treatment for four hours significantly decreased only the levels of *β*1 and *α*5 integrins' mRNA in the DU145 cells (*P* < 0.05). In conflict with our data, Engl et al. reported very low levels of *α*2 integrin mRNA in DU145 cells and CXCL12 only upregulated the level of *α*5 subunit when DU145 cells were stimulated with CXCL12 [26]. FACS scan analysis (Figure 8) was also performed for quantitative assessment of *α*4, *α*5, and *β*1 integrins' surface expression in untreated and CXCL12-treated PC3 and DU145 cells. In this experiment, cells were treated with CXCL12 (200 ng/mL); the percentage of cells expressing *α*4, *α*5, and *β*1 integrin subunits were calculated based on the percentage of mean fluorescence cells (%MFC). In concordance with cell adhesion and mRNA expression studies, flow cytometry analysis showed no surface expression of *α*4 integrin subunit in PC3 and DU145 cells. These findings explain why PC3 and DU145 cells' adhesion on FN was not mediated by *α*4*β*1 integrin. This might be related to migratory behaviors of these PCa cells as several studies have reported the association of loss of integrin *α*4 with metastatic potential of melanoma, fibrosarcoma, cholangiocarcinoma, and gastric cancer cells [47, 48]. The *β*1 integrin surface expression was detected in PC3 cells, but not *α*5 subunit; CXCL12 treatment had no effect on expression level. This suggests that PC3 cell adhesion on FN is not mediated by *α*5*β*1 integrin although the inhibition of PC3 cell adhesion on FN by anti-*β*1 integrin (mAB 13) shows that other *β*1-containing integrins are involved. In contrast, DU145 cells showed surface expression of *α*5 and *β*1 integrins whereas CXCL12 decreased *α*5 surface expression significantly. CXCL12 also reduced *β*1 integrin surface expression; however this reduction was not significant (*P* < 0.1). The inhibitory effect of CXCL12 on surface expressions of *β*1 and *β*3 integrins had been previously reported in A498, a cell line derived from renal cell carcinoma [13]. These findings not only indicate the different patterns of integrin expressions in DU145 and PC3 cells, but also explain the role of CXCL12 in decreasing DU145 cell adhesion on FN. This may explain how the elevated plasma CXCL12 could affect directional cell migration through chemotaxis process.

## 4. Conclusion

In summary, our results show that CXCL12 alongside PSA may be used as a potential biomarker for discriminating PCa from BPH patients. The elevated CXCL12 may be correlated with poor prognosis and aggressiveness of prostate cancer. The effects of CXCL12 on the expression and/or activity of integrins along with their repertoire on cell surfaces may affect prostate cancer cell adhesion and the manner in which these cells spread in microenvironments containing FN and COL.

## Figures and Tables

**Table 1:** Plasma CXCL12 concentrations in the 3 groups analyzed.

	*n*	Median (ng/mL)	Mean (ng/mL) ± SD	Median age
Age-matched control	33	1.29 (0.9–1.65)	1.43 ± 0.29	73
BPH	40	1.48 (1–2.2)	1.51 ± 0.32	70
PCa	39	1.8 (1.3–3)	1.93 ± 0.47	71

**Table 2:** Mean and median plasma CXCL12 concentrations in subgroup H (Gleason score >7 including 4 + 3) were significantly higher than in subgroup L (Gleason score >7 including 4 + 3).

	*n*	Median (ng/mL)	Mean (ng/mL) ± SD
PCa subgroup H	19	2.269	2.24 ± 0.44
PCa subgroup L	20	1.626	1.6 ± 0.2
